# Association of composite dietary antioxidant index with depression and all-cause mortality in middle-aged and elderly population

**DOI:** 10.1038/s41598-024-60322-0

**Published:** 2024-04-29

**Authors:** Juanjuan Luo, Xiying Xu, Yiyan Sun, Xixue Lu, Leiyong Zhao

**Affiliations:** 1 University City Hospital, Affiliated Hospital of Shandong University of Chinese Medicine, Jinan, China; 2 Affiliated Hospital of Shandong University of Traditional Chinese Medicine, Jinan, China; 3 Shandong University of Traditional Chinese Medicine, Jinan, China; 4 Neck Shoulder and Lumbocrural Pain Hospital of Shandong First Medical University, Shandong First Medical University (Shandong Academy of Medical Sciences), Jinan, China

**Keywords:** Association, CDAI, Depression, All-cause mortality, Stratified analysis, Psychology, Diseases, Medical research, Risk factors

## Abstract

Current research has shown an increasing acceptance of interventions for depression through dietary modifications. However, whether composite dietary antioxidant index (CDAI) is associated with depression and all-cause mortality in middle-aged and elderly population remains unknown. This study aimed to explore those associations in American middle-aged and elderly population. Weighted logistic regression models and weighted Cox proportional hazard regression models were used to assess the association of CDAI, covariates, depression, and all-cause mortality, respectively. The stability of the results was also determined by a linear trend test based on CDAI quintiles. Restricted cubic spline curves were employed to test for non-linear relationships. In the model adjusted for all covariates, significant associations were found with the ORs (95% CI) for CDAI and depression [0.77 (0.67, 0.89)] and the HRs (95% CI) for CDAI with all-cause mortality[0.91 (0.83, 1.00)]. Upon conducting restricted cubic spline curves, we found that the association between CDAI and depression was linear, whereas the association between CDAI and all-cause mortality was non-linear with an inflection point of -0.19. Statistical significance was only found before the inflection point. In this study of middle-aged and elderly Americans, CDAI was linearly negatively associated with depression and non-linearly negatively associated with all-cause mortality.

## Introduction

Depression is an affective disorder characterized by severe and persistent symptoms of mood, cognition, and physical state, such as low self-esteem, anhedonia, lack of energy, despondency, irritability, and fatigue^1,2^. Severe depression can even lead to suicide and is one of the most common mental illnesses, significantly affecting cognitive functioning, quality of life, and overall health^3^. Moreover, depression has become a serious public health issue, increasing social and economic burdens and the risk of death^4^. Epidemiological studies indicate that more than 350 million people worldwide suffer from depression^5^. The World Health Organization (WHO) predicts that by 2030, major depression will become the leading cause of global disease burden^6^. Research has found that the onset and severity of depression are not only influenced by genetic factors but also by environmental and dietary factors^7–9^.

Dietary interventions for chronic diseases have become increasingly recognized. The Mediterranean diet, based on a high intake of vegetables and fruit, fish, grains, legumes, and olive oil, has been shown to offer protection against heart disease, diabetes, and cardiovascular disease. It also prevents cerebrovascular damage, thereby reducing the risk of stroke, memory loss, and all-cause mortality^10,11^. The dietary approach to stop hypertension (DASH) diet promotes a diet high in potassium, low in sodium, magnesium, and calcium, and high in dietary fiber and unsaturated fatty acids, which is well proven to be effective in lowering blood pressure^12^. The flexible vegetarian diet that emphasizes increased vegetable consumption and reduced meat intake, is very effective in weight loss^13^. Anti-inflammatory diets focusing on fish, fruits, and vegetables have also been demonstrated a suppressive effect on the risk of Alzheimer's disease, Parkinson's disease, atherosclerosis, heart disease, and muscle wasting^14–17^. Furthermore, a meta-analysis showed that the addition of the antioxidant vitamin E to the treatment of depression and anxiety might be beneficial^18^.

The composite dietary antioxidant index (CDAI) is a measurement of individual antioxidant profiles derived from dietary combinations developed to assess and reflect the overall impact of dietary antioxidants on health^19^. Initially, research on the CDAI predominantly addressed cancer; however, its relevance has increasingly expanded into psychiatry in recent years^20,21^. An Iranian study highlighted that the dietary antioxidant index was associated with a reduced incidence of depression and anxiety in young women. However, the association of CDAI with depression and all-cause mortality in the middle-aged and elderly, a population with a high prevalence of depression and death, has still not been explored. To address this gap, we extracted data from the National Health and Nutrition Examination Survey (NHANES) for six cycles (2007–2018) to investigate these associations.

## Methods

### Study population

**Figure 1:**
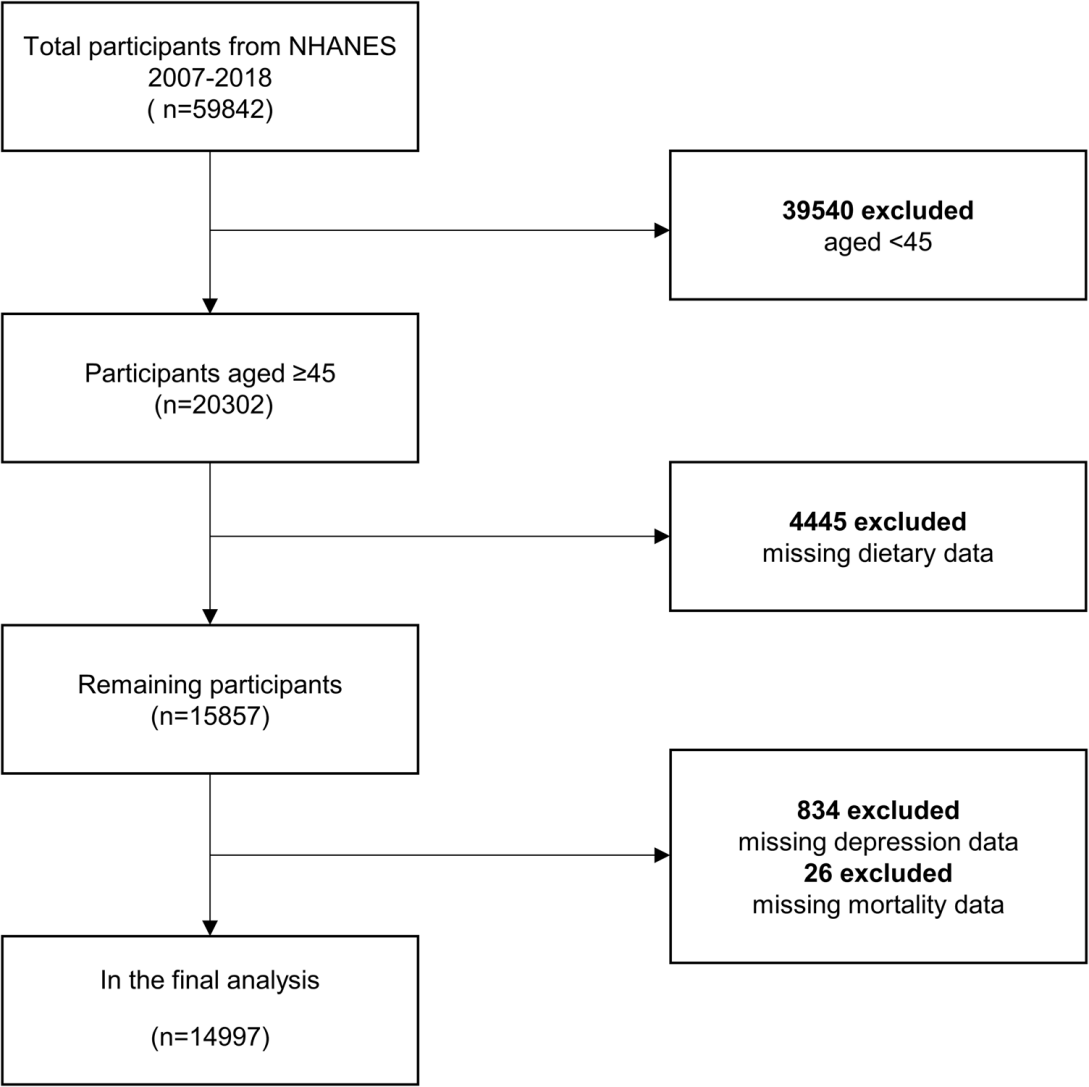
Flow chart of sample selection.

NHANES, initiated in 1999 by the Centers for Disease Control and Prevention, is a biennial observational survey that assesses the health and nutritional status of the non-institutionalized US population. It combines interviews with physical examinations to gather comprehensive data on demographics, socioeconomics, dietary habits, and health status. Medical assessments, physiological measurements, and laboratory examinations were conducted by highly qualified medical specialists. The NHANES protocol was approved by the National Center for Health Statistics of the Institutional Review Board, and all participants' informed consent was obtained. For this study, data from six cycles were analyzed, encompassing 14,997 individuals after excluding those who were: (1) younger than 45 years (n = 39,540); (2) missing CDAI data (n = 445); (3) missing depression data (n = 834), missing mortality data(n = 26) (Fig. 1).

### Assessment of composite dietary antioxidant index

Dietary assessments were based on two 24-h recall interviews conducted by trained dietary interviewers. The first recall was carried out in person using a standardized protocol during the medical examination at the mobile screening center. The second recall was conducted by telephone within 3 to 10 days of the first recall. Nutritional intakes were calculated based on food intake through the use of a revised nutritional database which translates each individual's food intake into nutrients. We calculated CDAI for all subjects from six dietary minerals and vitamins (Mn, Se, Zn, Vitamins A, C, and E) using Wright's recommended measures^22^. The detailed calculation formula was as follows:

$$CDAI = \mathop \sum \limits_{{{\text{i}} = 1}}^{{{\text{n}} = 6}} \frac{{{\text{Individual Intake}} - {\text{Mean}}}}{{{\text{SD}}}}$$

### Assessment of depression and all-cause mortality

The diagnosis of depression was determined using the Patient Health Questionnaire-9 (PHQ-9) scores, with each item being scored on a scale of 0–3 and a total score range of 0–27. A PHQ-9 score of 10 or above is considered indicative of depressive symptoms, offering a good balance between sensitivity and specificity for depression diagnosis. This determination has been widely used in cross-sectional studies of depression^23–25^. We extracted mortality data from the National Death Index (NDI) death certificate records, from which the information on mortality status and follow-up time (in months) was collected from the date of participation to the end of the follow-up period (December 31, 2019).

### Covariates

Referring to previous literature, we included covariates potentially influencing CDAI, depression, and all-cause mortality. Demographic characteristics such as age, gender, race, education level, marital status, and income-to-poverty ratio were included. Health-related behaviors included smoking status, vigorous recreational activity, drinking status, and medical co-morbidity variables (CVD, stroke, hypertension, diabetes, and cancer) were also collected. Biochemical and physical examinations consisted of C-reactive protein (CRP), body mass index (BMI), and waist. Additionally, the 24-h dietary recall interviews were utilized to obtain energy intake.

### Statistical analysis

All statistical analyses were performed in R version 4.2.0 with the "Survey" package after considering the complex sampling design. All statistical tests were two-tailed, with the statistical significance set at *p* < 0.05. In the baseline population characteristics, we divided the study population into five groups based on quintiles of the CDAI. Continuous variables were expressed as means ± standard errors, and categorical variables were presented as percentages. The weighted linear regression method for continuous variables and the weighted chi-square test for categorical variables were used in the comparison of baseline characteristics. Given that depression and all-cause mortality were both dichotomous variables, weighted logistic regression models and weighted Cox proportional hazard regression models were used to assess the association of CDAI and covariates, depression, and all-cause mortality, respectively. The results of the weighted logistic regression models were expressed as odds ratios (ORs) and 95% confidence intervals (CIs), and for Cox proportional hazard regression models as hazard ratios (HRs) and 95% confidence intervals (CIs).

To exclude the influence of confounding factors, three models were constructed to explore the associations. Model 1 performed the analysis without adjustment for any covariates. Model 2 adjusted for age, sex, and race, and Model 3 adjusted for all covariates listed in Table 1. The robustness of the results was further evaluated by a linear trend test based on CDAI quintiles. Restricted cubic spline curves were employed to test for non-linear relationships between the outcome variable and exposure factors. Upon finding the non-linear relationship, the inflection point was calculated by a recursive algorithm, and a two-piecewise linear regression model was constructed. Stratified analyses and interactions were utilized to explore the characteristics of the relationship between CDAI with depression and all-cause mortality across different population subgroups.

**Table 1:** Characteristics of the study population.

	Overall (n = 14,997)	Q1 (n = 3000)	Q2 (n = 2999)	Q3 (n = 2999)	Q4 (n = 2998)	Q5 (n = 3001)	*P*-value
Age (years)	60.61 ± 10.38	61.74 ± 10.62	61.24 ± 10.75	60.92 ± 10.43	60.57 ± 10.24	59.04 ± 9.78	< 0.0001
Sex (%)							< 0.0001
Men	46.22	27.65	33.79	43.39	50.75	67.81	
Women	53.78	72.35	66.21	56.61	49.25	32.19	
Race/ethnicity (%)							< 0.0001
Mexican American	5.04	5.48	5.67	4.91	4.54	4.83	
White	75.62	67.82	72.40	77.02	78.84	79.41	
Black	9.67	15.85	11.88	8.45	7.28	6.92	
Other	9.66	10.85	10.04	9.63	9.34	8.85	
Education level (%)							< 0.0001
Less than high school	5.06	8.58	6.30	4.81	3.62	3.15	
High school	33.87	45.48	40.31	33.64	28.50	25.80	
More than high school	61.07	45.93	53.39	61.55	67.88	71.05	
Marital status (%)							< 0.0001
Married /Living with partner	67.62	57.18	65.04	68.45	71.76	72.41	
Divorced/separated/widowed	26.03	35.36	29.60	24.99	22.90	20.42	
Never married	6.36	7.46	5.36	6.56	5.33	7.17	
Smoking status (%)							< 0.0001
Never	15.60	23.93	17.62	14.24	12.04	12.71	
Former	32.69	27.97	29.72	32.52	36.82	34.58	
Current	51.71	48.11	52.66	53.24	51.14	52.70	
Drinking status (%)							< 0.0001
Never	11.33	16.45	13.40	11.11	9.57	8.02	
Former	17.36	22.64	18.31	15.57	15.94	15.94	
Current	71.31	60.91	68.29	73.32	74.49	76.04	
Vigorous recreational activities (%)							< 0.0001
Yes	16.31	7.80	12.09	14.77	18.76	24.89	
No	83.69	92.20	87.91	85.23	81.24	75.11	
Cancer (%)							0.0330
Yes	17.31	17.07	16.18	19.10	16.65	17.39	
No	82.69	82.93	83.82	80.90	83.35	82.61	
CVD (%)							< 0.0001
Yes	14.50	18.49	15.57	14.53	14.23	11.01	
No	85.50	81.51	84.43	85.47	85.77	88.99	
Stroke (%)							< 0.0001
Yes	4.82	7.68	5.31	4.12	4.76	3.07	
No	95.18	92.32	94.69	95.88	95.24	96.93	
Hypertension (%)							< 0.0001
Yes	55.45	58.83	59.17	56.12	53.66	51.15	
No	44.55	41.17	40.83	43.88	46.34	48.85	
Diabetes (%)							< 0.0001
Yes	17.17	20.45	18.13	17.71	16.70	13.99	
No	82.83	79.55	81.87	82.29	83.30	86.01	
Depression (%)							< 0.0001
Yes	7.98	13.14	9.43	8.09	5.56	5.36	
No	92.02	86.86	90.57	91.91	94.44	94.64	
All-cause mortality(%)							< 0.0001
Yes	10.71	14.35	12.09	10.99	8.84	8.55	
No	89.29	85.65	87.91	89.01	91.16	91.45	
Income-to-poverty ratio	3.27 ± 1.60	2.64 ± 1.59	2.96 ± 1.58	3.36 ± 1.58	3.55 ± 1.53	3.61 ± 1.54	< 0.0001
BMI (kg/m^2^)	29.57 ± 6.60	29.81 ± 6.83	29.69 ± 6.51	29.72 ± 6.73	29.38 ± 6.26	29.33 ± 6.67	0.0127
Waist (cm)	102.27 ± 15.43	101.69 ± 15.15	101.70 ± 14.96	102.30 ± 15.52	102.43 ± 15.47	102.92 ± 15.82	0.0110
Energy intake (kcal)	1986.12 ± 732.59	1231.67 ± 384.05	1617.87 ± 397.22	1892.91 ± 457.63	2169.18 ± 518.74	2735.07 ± 751.75	< 0.0001
CRP (mg/dl)	2.38 ± 5.95	3.53 ± 8.59	2.46 ± 4.30	2.50 ± 7.21	2.18 ± 4.97	1.56 ± 3.76	< 0.0001

*CVD* cardiovascular disease; *BMI* body mass index; *CRP* c-reactive protein.

### Ethical approval

The NHANES protocol was approved by the National Center for Health Statistics of the Institutional Review Board, and all participants' informed consent was obtained. All methods were carried out in accordance with relevant guidelines and regulations (declarations of Helsinki).

## Results

### Characteristics of the study population

In the baseline population of Table 1, men accounted for 46.22%, and women for 53.78%. The proportions of Mexican American, White, and Black were 5.04%, 75.6%, and 9.67%, respectively. Additionally, 61.07% of participants had a high school education or higher. Analysis revealed that individuals with higher CDAI were younger, more likely to be men, and white, with higher levels of education and income-to-poverty ratio. These individuals also reported higher energy intake, more frequent engagement in recreational activities, and exhibited lower risks of hypertension, diabetes, depression, and all-cause mortality, alongside reduced C-reactive protein levels.

### Univariate analysis

The results of the univariate analysis are presented in Table 2. Women had a higher risk of depression and a lower risk of all-cause mortality compared to men. Depression showed a negative association with income-to-poverty ratio, vigorous recreational activities, CVD, stroke, hypertension, diabetes, BMI, waist, and CRP, and smoking status was found to be positively associated with depression. The covariates positively associated with all-cause mortality included age, cancer, CVD, stroke, hypertension, diabetes, waist, and energy intake.

**Table 2:** Univariate analyses.

	Depression		All -cause mortality	
	OR (95% CI)	*p*-value	HR (95% CI)	*p*-value
Age	0.98 (0.97, 0.99)	< 0.0001	1.11 (1.10, 1.11)	< 0.0001
Sex				
Male	1.0		1.0	
Female	1.60 (1.34, 1.92)	< 0.0001	0.76 (0.69, 0.85)	< 0.0001
Race/ethnicity				
Mexican American	1.0		1.0	
White	0.69 (0.56, 0.85)	0.0007	1.83 (1.37, 2.44)	0.0001
Black	0.88 (0.71, 1.10)	0.2719	1.70 (1.26, 2.30)	0.0009
Other	1.11 (0.85, 1.45)	0.4563	1.00 (0.69, 1.43)	0.9873
Education level				
Less than high school	1.0		1.0	
High school	0.71 (0.60, 0.85)	0.0002	0.65 (0.54, 0.78)	< 0.0001
More than high school	0.36 (0.29, 0.45)	< 0.0001	0.35 (0.27, 0.44)	< 0.0001
Marital status				
Married/Living with partner	1.0		1.0	
Divorced/separated/widowed	2.14 (1.81, 2.53)	< 0.0001	2.15 (1.92, 2.41)	< 0.0001
Never married	2.23 (1.73, 2.88)	< 0.0001	1.37 (1.06, 1.77)	0.0190
Smoking status				
Current	1.0		1.0	
Former	0.39 (0.32, 0.48)	< 0.0001	1.06 (0.90, 1.26)	0.4844
Never	0.29 (0.24, 0.34)	< 0.0001	0.59 (0.50, 0.69)	< 0.0001
Drinking status				
Current	1.0	< 0.0001	1.0	
Former	2.12 (1.60, 2.80)		1.66 (1.38, 2.00)	< 0.0001
Never	1.04 (0.84, 1.29)	0.7203	0.51 (0.41, 0.63)	< 0.0001
Vigorous recreational activities				
Yes	1.0		1.0	
No	5.89 (4.04, 8.59)	< 0.0001	4.35 (3.25, 5.82)	< 0.0001
Cancer				
Yes	1.0		1.0	
No	1.11 (0.88, 1.40)	0.3880	0.46 (0.42, 0.52)	< 0.0001
CVD				
Yes	1.0		1.0	
No	0.47 (0.39, 0.56)	< 0.0001	0.25 (0.22, 0.30)	< 0.0001
Stroke				
Yes	1.0		1.0	
No	0.38 (0.29, 0.50)	< 0.0001	0.26 (0.21, 0.33)	< 0.0001
Hypertension				
Yes	1.0		1.0	
No	0.63 (0.53, 0.75)	< 0.0001	0.39 (0.34, 0.44)	< 0.0001
Diabetes				
Yes	1.0		1.0	
No	0.59 (0.50, 0.71)	< 0.0001	0.47 (0.42, 0.54)	< 0.0001
Income-to-poverty ratio	0.65 (0.60, 0.69)	< 0.0001	0.72 (0.68, 0.75)	< 0.0001
BMI	1.04 (1.03, 1.05)	< 0.0001	0.99 (0.98, 1.00)	0.2975
Waist	1.01 (1.01, 1.02)	< 0.0001	1.01 (1.00, 1.01)	0.0018
Energy intake	1.00 (1.00, 1.00)	0.0002	1.00 (1.00, 1.00)	< 0.0001
CRP	1.02 (1.01, 1.03)	< 0.0001	0.97 (0.93, 1.02)	0.2648

CVD, cardiovascular disease; BMI, body mass index; CRP, c-reactive protein.

### Association between CDAI and depression and all-cause mortality

**Figure 2:**
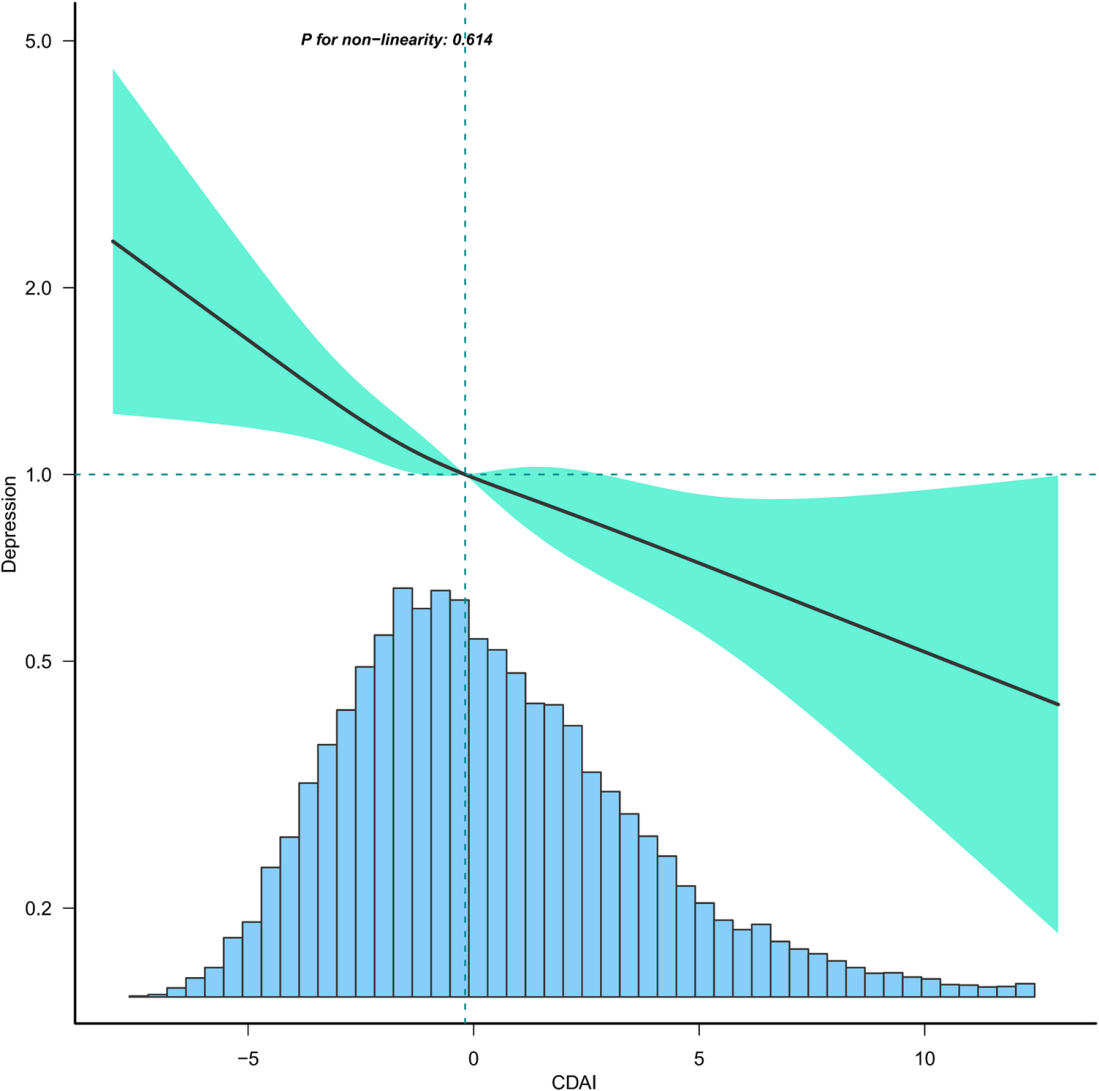
Association between CDAI and depression in middle-aged and elderly population. Age, sex, race, education level, income-to-poverty ratio, marital status, smoking status, drinking status, vigorous recreational activity, BMI, waist, stroke, diabetes, CVD, cancer, hypertension, energy intake, and CRP were adjusted.

**Figure 3:**
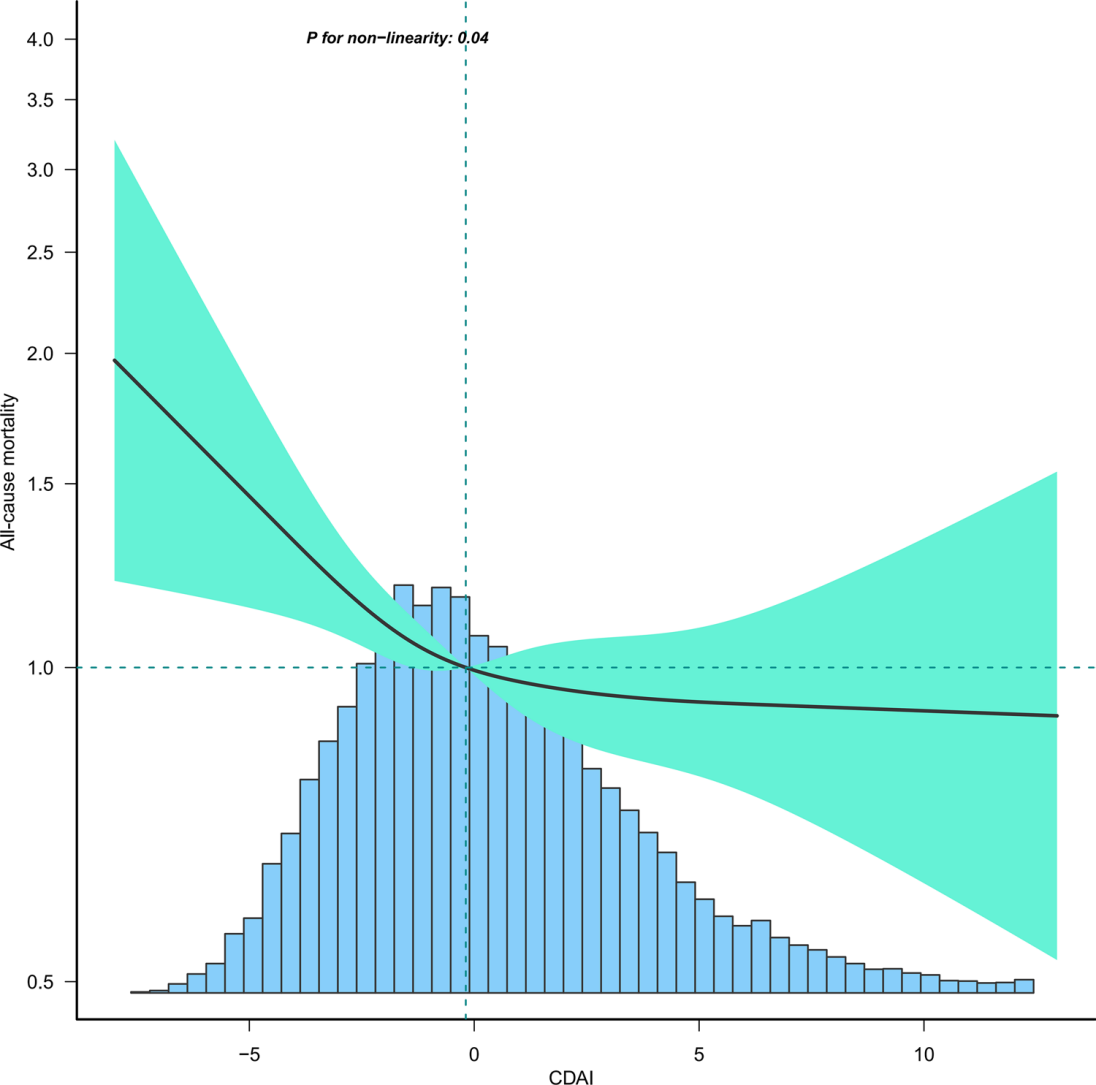
Association between CDAI and all-cause mortality in middle-aged and elderly population. Age, sex, race, education level, income-to-poverty ratio, marital status, smoking status,drinking status, vigorous recreational activity, BMI, waist, stroke, diabetes, CVD, cancer, hypertension, energy intake, and CRP were adjusted.

As shown in Table 3, the initial unadjusted analysis (Model 1) revealed that CADI was negatively associated with depression [0.74 (0.70, 0.79)] and all-cause mortality [0.84 (0.80, 0.88)]. This negative association persisted in regression models adjusted for all variables, with ORs for CDAI with depression at 0.77 (95% CI: 0.67, 0.89) and HRs for CDAI with all-cause mortality at 0.91 (95% CI: 0.83, 1.00). Comparing against the lowest quartile of CDAI (Q1) as the reference, higher CDAI was associated with a lower risk of depression, with ORs (95% CI) of 0.80 (0.61, 1.04), 0.67 (0.52, 0.87), 0.65 (0.48, 0.88), and 0.52 (0.36, 0.77). A similar trend was observed for all-cause mortality risk, with HRs (95% CI) of 0.87 (0.72, 1.05), 0.82 (0.69, 0.98), 0.77 (0.63, 0.96), and 0.75 (0.58, 0.96). Subsequent analysis using restricted cubic spline curves indicated a linear association between CDAI and depression (Fig. 2), whereas the relationship with all-cause mortality was non-linear with an inflection point of −0.19 (Fig. 3). This association was statistically significant only before this inflection point [0.71 (0.56, 0.89)] (Table 4).

**Table 3:** Association of composite dietary antioxidant index with depression and all-cause mortality in middle-aged and elderly population.

	Model 1 OR (95% CI)	*P*-value	Model 2 OR (95% CI)	*P*-value	Model 3 OR (95% CI)	*P*-value
Depression						
CDAI..Z score	0.74 (0.70, 0.79)	< 0.0001	0.75 (0.71, 0.80)	< 0.0001	0.77 (0.67, 0.89)	0.0003
Q1	1.0		1.0		1.0	
Q2	0.72 (0.62, 0.84)	< 0.0001	0.72 (0.61, 0.84)	< 0.0001	0.80 (0.61, 1.04)	0.0950
Q3	0.67 (0.57, 0.78)	< 0.0001	0.67 (0.57, 0.79)	< 0.0001	0.67 (0.52, 0.87)	0.0021
Q4	0.49 (0.41, 0.59)	< 0.0001	0.50 (0.42, 0.60)	< 0.0001	0.65 (0.48, 0.88)	0.0059
Q5	0.44 (0.37, 0.53)	< 0.0001	0.46 (0.38, 0.55)	< 0.0001	0.52 (0.36, 0.77)	0.0009
P for trend	< 0.0001		< 0.0001		0.0031	

	Model 1 HR (95% CI)	*P*-value	Model 2 HR (95% CI)	*P*-value	Model 3 HR (95% CI)	*P*-value
All-cause mortality						
CDAI..Z score	0.84 (0.80, 0.88)	< 0.0001	0.84 (0.80, 0.88)	< 0.0001	0.91 (0.83, 1.00)	0.0419
Q1	1.0		1.0		1.0	
Q2	0.87 (0.77, 0.99)	0.0316	0.81 (0.71, 0.92)	0.0009	0.87 (0.72, 1.05)	0.1456
Q3	0.84 (0.74, 0.95)	0.0049	0.80 (0.71, 0.91)	0.0006	0.82 (0.69, 0.98)	0.0305
Q4	0.69 (0.60, 0.79)	< 0.0001	0.64 (0.56, 0.74)	< 0.0001	0.77 (0.63, 0.96)	0.0184
Q5	0.62 (0.54, 0.71)	< 0.0001	0.62 (0.54, 0.71)	< 0.0001	0.75 (0.58, 0.96)	0.0238
P for trend	< 0.0001		< 0.0001		0.0600	

Model 1: No covariates were adjusted.

Model 2: Age, sex, race were adjusted.

Model 3: Age, sex, race, education level, income-to-poverty ratio, marital status, smoking status,drinking status, vigorous recreational activity, BMI, waist, stroke, diabetes, CVD, cancer, hypertension, energy intake, and CRP were adjusted.

**Table 4:** Threshold effect analysis of composite dietary antioxidant index on all-cause mortality in middle-aged and elderly population using a two-piecewise linear regression model.

All-cause mortality	Adjust HR (95% CI)	*P*-value
CDAI		
Fitting by standard linear model	0.91 (0.83, 1.00)	0.0419
Fitting by two-piecewise linear model		
Inflection point	− 0.19	
< −0.19	0.71 (0.56, 0.89)	0.0029
> −0.19	0.99 (0.96, 1.02)	0.4288
Log-likelihood ratio	0.021	

Age, sex, race, education level, income-to-poverty ratio, marital status, smoking status, drinking status, vigorous recreational activity, BMI, waist, stroke, diabetes, CVD, cancer, hypertension, energy intake, and CRP were adjusted.

### Subgroup analysis

To explore the disparities in the relationship between CDAI and depression and all-cause mortality in various subgroups, stratified analyses and interactions were conducted in the middle-aged and elderly population (Table 5), and significant differences were found in these relationships only in the smoking population (*P* interaction = 0.0343 and *P* interaction = 0.0399). The associations between CDAI and depression and all-cause mortality were 0.76 (0.59, 0.97) and 0.69 (0.54, 0.89) for former smokers. For those who had never smoked, the relationship was 0.64 (0.51, 0.80) and 0.64 (0.51, 0.80).

**Table 5:** Subgroup logistic regression analysis for the association between CDAI and outcomes in middle-aged and elderly population.

Subgroup	Depression		All -cause mortality	
	OR (95% CI)	*p* interaction	HR (95% CI)	*p* interaction
Sex		0.3673		0.5397
Male	0.83 (0.67, 1.02)		0.88 (0.79, 0.99)	
Female	0.73 (0.61, 0.88)		0.93 (0.80, 1.09)	
Race/ethnicity		0.1954		0.0986
Mexican American	0.54 (0.36, 0.81)		0.73 (0.47, 1.13)	
White	0.77 (0.64, 0.94)		0.90 (0.80, 1.00)	
Black	0.87 (0.63, 1.21)		0.79 (0.61, 1.02)	
Other	0.91 (0.67, 1.24)		1.27 (0.93, 1.75)	
Education		0.4431		0.2725
Less than high school	0.61 (0.41, 0.92)		1.02 (0.79, 1.31)	
High school	0.80 (0.65, 0.98)		0.94 (0.82, 1.08)	
More than high school	0.81 (0.66, 1.00)		0.94 (0.82, 1.08)	
Marital status		0.8056		0.3982
Married/Living with partner	0.77 (0.63, 0.93)		0.86 (0.76, 0.98)	
Divorced/separated/widowed	0.81 (0.66, 1.00)		0.98 (0.85, 1.14)	
Never married	0.90 (0.56, 1.44)		0.94 (0.63, 1.41)	
Vigorous recreational activities		0.2208		0.2244
Yes	1.05 (0.64, 1.72)		1.13 (0.77, 1.65)	
No	0.75 (0.65, 0.87)		0.88 (0.80, 0.97)	
Smoking status		0.0343		0.0399
Current	0.99 (0.78, 1.26)		0.86 (0.69, 1.06)	
Former	0.76 (0.59, 0.97)		0.69 (0.54, 0.89)	
Never	0.64 (0.51, 0.80)		0.57 (0.46, 0.72)	
Drinking status		0.3943		0.8828
Current	0.60 (0.39, 0.93)		0.96 (0.75, 1.22)	
Former	0.75 (0.57, 0.97)		0.89 (0.76, 1.04)	
Never	0.82 (0.69, 0.97)		0.90 (0.79, 1.03)	
Cancer		0.0740		0.9328
Yes	0.81 (0.70, 0.94)		0.90 (0.80, 1.00)	
No	0.56 (0.38, 0.83)		0.91 (0.76, 1.08)	
Stroke		0.4006		0.8727
Yes	0.64 (0.40, 1.02)		0.88 (0.69, 1.12)	
No	0.79 (0.68, 0.91)		0.90 (0.81, 0.99)	
CVD		0.7686		0.9631
Yes	0.74 (0.56, 0.98)		0.90 (0.77, 1.04)	
No	0.78 (0.67, 0.92)		0.90 (0.80, 1.01)	
Diabetes		0.5305		0.3756
Yes	0.85 (0.56, 1.27)		0.84 (0.70, 1.01)	
No	0.77 (0.63, 0.93)		0.93 (0.83, 1.03)	
Hypertension		0.5406		0.4842
Yes	0.80 (0.67, 0.95)		0.89 (0.80, 0.99)	
No	0.73 (0.58, 0.92)		0.96 (0.80, 1.14)	

In the subgroup analysis stratified by sex, race, education, marital status, vigorous recreational activities, smoking status, drinking status, stroke, CVD, diabetes, hypertension, and cancer, the model is not adjusted for sex, race, education, marital status, vigorous recreational activities, smoking status, drinking status, stroke, CVD, diabetes, hypertension, and cancer, respectively.

## Discussion

In the observational study among middle-aged and elderly Americans, higher CDAI was associated with a lower risk of depression and all-cause mortality. Further restricted cubic spline curves revealed a linear association of CDAI with depression and a non-linear association of CDAI with all-cause mortality. The inflection point was detected with the relationship being statistically significant only before the inflection point. To explore whether there were differences in the relationships among various subgroups, we conducted stratified analyses. To the best of our knowledge, this investigation represented the inaugural attempt to examine the association of CDAI with depression and all-cause mortality in a middle-aged and elderly population, and the most detailed subgroup analysis was performed.

The association between dietary antioxidants and depression and all-cause mortality has received increasing attention in recent years. Evidence suggested that only antioxidants derived from food sources, not dietary supplements, could offer protective benefits against depression^26^. Observational meta-analyses have shown that the intakes of vitamins A, C, and E were inversely related to the incidence of depression^27,28^. A cross-sectional study in the United States indicated that higher zinc intake was associated with a lower risk of depression^29^. Interestingly, a study from Brazilian Farmers showed a non-linear negative correlation between dietary selenium intake and depression^30^. Similarly, a study on the general American population found a non-linear negative relationship between CDAI and depression^21^. However, both our study and the study of Iranian adolescent girls revealed that the relationship between CDAI and depression was linearly negative^31^. Moreover, the findings on the relationship between dietary antioxidants and all-cause mortality were often inconsistent. A Korean study suggested that dietary zinc intake was associated with lower all-cause mortality^32^, contradicting a study in China, which found a positive correlation between zinc intake and all-cause mortality^33^. Additionally, studies in China and the United States have shown that selenium intake was linked to reduced all-cause mortality^34,35^. An elderly epidemiological study indicated that supplementing with vitamins C and E could lower the risk of all-cause mortality^36^. However, a cohort study from the UK Biobank found no significant association between antioxidant use and all-cause mortality^37^. In contrast, a cohort study of American cancer survivors revealed a significantly negative association between CDAI and the risk of mortality^38^. Our research identified a non-linear relationship between CDAI and all-cause mortality, whereas a study on the general US population indicated a linear relationship between CDAI and all-cause mortality^39^. Age and geographic differences may account for the inconsistent relationship of dietary antioxidants with depression and all-cause mortality. People in different geographical areas have different dietary habits, which leads to differences in antioxidant intake. In addition, middle-aged and elderly people tend to decline in physical function, which may affect the absorption and utilization of dietary antioxidants, consequently causing inconsistencies in the results of the studies.

Although the specific mechanisms linking CDAI to depression and all-cause mortality in middle-aged and elderly population are still not well understood, some possible molecular mechanisms have been suggested in some studies. The first is oxidative stress. Depressed people often experience oxidative stress, characterized by an imbalance between oxygen free radicals and antioxidant substances in the body^40^. Oxygen free radicals are harmful molecules that interact with other molecules in the body and cause oxidative damage to cells and tissues^41^. Consuming a diet rich in antioxidants has been shown to lower oxidative stress and protect cells from damage caused by free radicals,thereby alleviating symptoms of depression^42^. Studies have shown that vitamins C and E can trap free radicals and protect molecules such as membrane fatty acids, cholesterol, and proteins from oxidative damage, thus reducing the risk of developing chronic diseases such as cancer, cardiovascular disease, and diabetes^43,44^. Additionally, appropriate exogenous antioxidants can decrease the risk of mortality in diabetic population by balancing oxidative stress reactions^45^. The second is chronic inflammation: Strong evidence suggests that neuroinflammation can promote the emergence and development of depression^46,47^. The antioxidant properties of the diet are capable of diminishing the neuroinflammatory response by modulating the levels of inflammatory factors. Some studies have shown that dietary zinc, vitamin A, and E can lower levels of inflammatory factors such as interleukin-6 (IL-6), tumor necrosis factor-alpha (TNF-α), and interferon-gamma (IFN-γ), consequently reducing the impact of neuroinflammation on depression^48^. Other studies have shown that chronic inflammation is strongly associated with the development and progression of a variety of diseases, including cardiovascular disease, diabetes, obesity, and cancer, as well as being associated with an increased risk of all-cause mortality^49,50^. Antioxidant diets could significantly reduce inflammatory markers, improve chronic disease states, and reduce mortality risk^51–53^. The third is neuroplasticity. Depressed patients often experience impaired neuroplasticity and reduced levels of brain-derived neurotrophic factor (BDNF). BDNF is known to be essential for the growth, differentiation, and survival of nerve cells, and BDNF also promotes neuronal connectivity and the formation of neural networks, which is a key factor in neuroplasticity^54^. Antioxidant diets can affect the production and release of neurotrophic factors such as neurotrophic factor (NGF) and BDNF^55^. These neurotrophic factors promote neuronal proliferation and synaptic plasticity, helping to maintain and enhance neurological health and thus improving depressive symptoms^56^. The fourth is neurotransmitters. Neurotransmitters such as serotonin, dopamine, and norepinephrine, are critical in regulating mood and depression^57^. Antioxidants contribute to the protection of nerve cells from damage and improve neurotransmitter function, which can help prevent and treat depression. Specifically, the antioxidant vitamin C has been found to promote the release of neurotransmitters such as dopamine, norepinephrine, and 5-hydroxytryptamine from neurons, increasing the levels of neurotransmitters that influence communication between neurons^58^. The fifth is the gut microbiota. Imbalances in the gut microbiota have been shown to be strongly associated with the development of depression and adverse outcomes of various diseases^59^. Compared with healthy individuals, the gut microbial composition of depressed patients changed, particularly in microbial diversity and relative abundance of specific bacterial taxa^60,61^. In hemodialysis patients, the microbial diversity of non-survivors was significantly lower than that of survivors, especially in terms of amber fungi and anaerobic bacteria^62^. Studies have indicated that dietary antioxidant micronutrients can alleviate depressive symptoms and extend the life span of patients by modulating the abundance of the gut microbiota^63,64^.

Subgroup analyses were conducted to better explore differences among subgroups. Notably, in stratified analyses incorporating demographic characteristics, lifestyle factors, and comorbidities, a statistically significant interaction emerged specifically regarding smoking status. This finding indicated that those who had never smoked benefited more from an antioxidant-rich diet than those who had smoked and were still smoking. Studies have shown that smoking can lead to an increase in lipid peroxidation products and extracellular matrix protein degradation products, chemotaxis of activated neutrophils and macrophages, increased levels of circulating pro-inflammatory markers and oxidative stress markers, including reactive oxygen species (ROS) levels, superoxide dismutase (SOD), and glutathione peroxidase (GPx), along with a decrease in antioxidant levels^65–68^. Abnormal levels of inflammation and antioxidants can exacerbate the onset of depression and death^69,70^. Thus, in people who have never smoked, an antioxidant diet may better reduce the risk of depression and all-cause mortality.

Our study has several strengths. First, NHANES used standardized methods for data collection and analysis, coupled with stringent quality control of the data to ensure accuracy and reliability. Second, NHANES censuses thousands of participants from all over the country each year, enabling a large sample size and representativeness that provide a wide range of data on the health status of the US population. Third, we performed restricted cubic spline analyses to assess the dose–response relationship between CDAI and the risk of depression and all-cause mortality. Fourth, as global aging increases, it is important to study a large sample of data on middle-aged and elderly adults for health guidance and public health policy development in older populations. Fifth, a more comprehensive inclusion of risk factors affecting depression and all-cause mortality makes the study results more credible. There are also some limitations to this study. First, the observational study designs could only indicate that higher CDAI was associated with a lower risk of depression and all-cause mortality in middle-aged and older populations, whereas no conclusions about causality could be drawn. Second, given that the dietary data were obtained through follow-up interviews, and considering potential memory loss in the middle-aged and elderly population with depressive disorders, recall bias was an unavoidable limitation. Third, the dietary data were extracted from only two follow-up visits and therefore may not accurately reflect the typical dietary patterns of the subjects. Fourth, since this study was conducted on a middle-aged and older population in the United States, this limits the applicability of the findings to other demographics, and future research is necessary to examine the relevance of these findings in different international contexts.

## Conclusion

In this study of middle-aged and elderly Americans, CDAI was linearly negatively associated with depression and non-linearly negatively associated with all-cause mortality. The stratified analysis revealed differences between populations with different smoking statuses, and these findings may help public health authorities tailor policies to prevent and improve people's emotional states and increase longevity. Further basic research will be required to discover the molecular biological mechanisms underpinning these associations.

## Data Availability

The data that support the findings of this study are available at https://wwwn.cdc.gov/nchs/nhanes.
